# Awareness, treatment, control, and determinants of dyslipidemia among adults in China

**DOI:** 10.1038/s41598-021-89401-2

**Published:** 2021-05-12

**Authors:** Sampson Opoku, Yong Gan, Emmanuel Addo Yobo, David Tenkorang-Twum, Wei Yue, Zhihong Wang, Zuxun Lu

**Affiliations:** 1 Department of Social Medicine and Health Management, School of Public Health, Tongji Medical College, Huazhong University of Science and Technology, No. 13 Hangkong Road, Wuhan, 430030 China; 2 Department of Medicine, SUNY Upstate Medical University, New York, USA; 3 Department of Adult Health, School of Nursing and Midwifery, University of Ghana, Accra, Ghana; 4 Department of Neurology, Tianjin Huanhu Hospital, Tianjin, China; 5 Department of Neurology, Shenzhen Second People’s Hospital, Shenzhen University, Shenzhen, Guangdong China

**Keywords:** Cardiology, Health care, Risk factors

## Abstract

Effective management of dyslipidemia is important. This study aimed to determine the awareness, treatment, control, and determinants of dyslipidemia in middle-aged and older Chinese adults in China. Using data from the 2015 China National Stroke Screening and Prevention Project (CNSSPP), a nationally representative sample of 135,403 Chinese adults aged 40 years or more were included in this analysis. Dyslipidemia was defined by the Third Report of the National Cholesterol Education Program Expert Panel on Detection, Evaluation, and Treatment of High Blood Cholesterol in Adults final report (NCEP-ATP III) and the 2016 Chinese guidelines for the management of dyslipidemia in adults. Models were constructed to adjust for subjects’ characteristics with bivariate and multivariable logistic regression analyses. Overall, 51.1% of the subjects were women. Sixty-four percent were aware of their condition, of whom 18.9% received treatment, and of whom 7.2% had adequately controlled dyslipidemia. Dyslipidemia treatment was higher in men from rural areas than their urban counterparts. The multivariable logistic regression models revealed that women, urban residents, and general obesity were positively related to awareness. Women, married respondents, and current drinkers had higher odds of treatment. Age group, overweight, general obesity, urban residence, and women were independent determinants of control. Dyslipidemia awareness rate was moderately high, but treatment and control rates were low. Results can be used to develop policies and health promotion strategies with special focus on middle-aged and older adults.

## Introduction

Cardiovascular diseases (CVDs) are the leading cause of morbidity and mortality globally and dyslipidemia is an established risk factor of CVDs such as atherosclerosis^1–3^. Dyslipidemia awareness, treatment, and control have been comprehensively studied among adults. Further, increased awareness, treatment and control of dyslipidemia have contributed to improved primary and secondary prevention of consequent cardiovascular events in developed countries^4^. Yet, the rates of dyslipidemia screening and treatment that have been reported across many low- and middle-income countries vary substantially^5^. China’s fast economic development has led to lifestyle changes, including increased tobacco consumption, unhealthy nutrition, and reduced physical activity, which in turn, have led to increased prevalence of dyslipidemia and other CVDs’ risk factors in the country^6^. Some previous studies on dyslipidemia among adults in China reported low awareness and management rates. For example, in 2011, Song et al. reported dyslipidemia awareness, treatment, and control rates among Chinese adults as 20.3, 14.4, and 4.9%, respectively^7^. Additionally, a prior national study reported high prevalence rates for total cholesterol (TC), low-density lipoprotein (LDL-C), and triglycerides (TG), but low awareness and treatment rates^8^. Further, in 2014, a systematic review on dyslipidemia epidemiology reported the following estimates: prevalence, 42%; awareness, 24.4%; treatment, 8.8%; and control, 4.3%^9^. With reference to the “rule of halves” framework^10^, it can be observed from the aforementioned results that the rates of dyslipidemia awareness, treatment, and control have been low. The ‘rule of halves’ is a proxy framework used to estimate indicators of unmet needs for chronic diseases and stipulates that: (1) about 50% of all diseases should be diagnosed, (2) of whom about 50% should receive treatment and, (3) of whom about 50% should achieve treatment targets. Meanwhile, earlier studies have demonstrated that ageing is a recognized lipid-related risk factor of CVDs, and an effective treatment and control of dyslipidemia among adults can help reduce consequent CVDs^11,12^. Additionally, improved awareness and positive lifestyle adoption among individuals with dyslipidemia are essential for effective disease management^13^. Hence, a recent estimate of dyslipidemia awareness and management among Chinese adults is needed to better evaluate the effectiveness of care and inform stakeholders on measures to improve disease management. However, studies on dyslipidemia awareness, treatment, and control and their determinants at the national level in China are limited. Therefore, we aimed to estimate the awareness, treatment, and control of dyslipidemia and their determinants among middle-aged and older Chinese adults using a nationwide survey data.

## Methods

### Study population and sampling

This cross-sectional study retrieved data from the 2015 survey of the China National Stroke Screening and Prevention Project (CNSSPP). This project is a nationwide program instituted by the Chinese government in 2011. The survey is conducted annually as a stroke surveillance program in the country. Details have been described elsewhere^14^. In brief, the survey involved Chinese adults aged 40 years or more. It used a two-stage stratified cluster sampling procedure. Firstly, 200 project areas were selected in proportion to the local population size and numbers of total counties. Secondly, an urban community and a rural village were determined as primary sampling units within each project area according to geographical location and suggestions from local hospitals. This cluster sampling method was implemented in each primary sampling unit. All individuals aged 40 years or above were recruited during the primary screening^14^. About 180,000 participants were randomly selected for further assessment for stroke risk factors such as dyslipidemia. Questionnaires completion and assessment were done in primary healthcare facilities.

### Data collection

Personnel were trained to collect data from participants using questionnaires. The questionnaires consisted of several domains, including socio-demographics, medical history, and lifestyle information. In this study, age was grouped into four categories: 40–49, 50–59, 60–69, and ≥ 70 years old)^15^. Ethnicity was categorized as ‘Han’ (majority ethnic group in China), and ‘other ethnicities’ (respondents who belong to the remaining ethnicities)^6^. Sex was categorized as either male or female, and marital status was grouped as either married or unmarried^16^. Area of residence was classified into urban or rural areas. An urban area was defined with the 12-component study-specific urbanization index. This criterion had been previously validated to capture the degree of urbanization in the study area (reliability across study waves [Cronbach’s Alpha]: 0.85–0.89; validity [correlation with official classification]; 0.75–0.78)^17^. Educational status was categorized as primary school or below, junior/middle school, senior/middle school, and college or above^18^. Current smoking was defined as smoking at least one cigarette per day in the last 3 months^6^. Current drinking was defined as drinking alcoholic beverages ≥ 1 per week for more than half a year^6^. Physical activity was defined as engaging in an activity at least three times per week for at least 30 min each episode, or engaging in heavy physical work^18^. Family history of dyslipidemia was defined on the basis of whether the respondent’s family (parents and siblings) had been diagnosed by a physician for dyslipidemia^19^.

Two geographical groupings were used to zone participants: (a) the northern and southern zones (according to the Huai River–Qin Mountains Line)^20^, and (b) the stroke and non-stroke belt zones. The stroke belt zone was defined based on stroke incidence in China, i.e., any region containing provinces that met the criteria for a region of high stroke incidence^21^.

Height and weight were measured with subjects in light clothing and not wearing shoes. A stadiometer was used to measure height to the nearest 0.1 cm (cm) in a standing position with closed feet, whereas weight was measured to the nearest 0.1 kilograms (kg) using a digital scale in a standing position. The body mass index (BMI) was calculated as the weight in kilograms divided by the height in meters squared (kg/m^2^) based on China’s Ministry of Health criteria^22^. BMI categories included: underweight, < 18.5 kg/m^2^; normal weight, ≥ 18.5 kg/m^2^ and < 24 kg/m^2^; overweight, ≥ 24 kg/m^2^ and < 28 kg/m^2^; and obesity, ≥ 28 kg/m^2^. Waist circumference (WC) was measured at the midpoint between the iliac crest and the lower rib to the nearest 0.1 cm. Central obesity was defined with the cutoffs points for Asians (˃ 90 cm in men and ˃ 80 cm in women)^23^.

### Laboratory assay

A standardized protocol was used to collect blood samples from participants at all research centers. Blood samples were drawn from subjects’ antecubital veins to measure TG, TC, LDL-C, and high-density lipoprotein cholesterol (HDL-C) values. The samples were collected in the morning after an 8-h overnight fast and transported to the same laboratory (Changchun Kingmed Centre for Clinical Laboratory Co Ltd.) under refrigeration, and then stored at − 20 °C.

### Criteria for assessment

Dyslipidemia was defined according to the Third Report of the National Cholesterol Education Program (NCEP) Expert Panel on Detection, Evaluation, and Treatment of High Blood Cholesterol in Adults final report (NCEP-ATP III)^24^. This categorization is the same as that of the latest 2016 Chinese guidelines for the prevention and treatment of dyslipidemia in adults^25^. Dyslipidemia was defined as having either or a combination of TG, LDL-C, HDL-C and TC representing ≥ 2.26, ≥ 4.14, < 1.04, and ≥ 6.22 mmol/l, respectively, or a current use of lipid-modifying medications^25^. Awareness of dyslipidemia was defined as a self-reported physician diagnosis of dyslipidemia or self-reported use of lipid-lowering medications within the population defined as having dyslipidemia. In addition, treatment of dyslipidemia was defined as using prescribed lipid-lowering medications to treat dyslipidemia among participants with dyslipidemia, and control of dyslipidemia was defined as having dyslipidemia and being treated with medications if the individual has TC < 6.22 mmol/l, LDL-C < 4.14 mmol/l, HDL-C ≥ 1.04 mmol/l and TG < 2.26 mmol/l^8,9,22^.

### Data analysis

The International Business Machine Statistical Package for Social Sciences (IBM SPSS) version 19.0 (SPSS Inc, Chicago, Ill) software was used for all analyses. To reduce measurement errors, individuals with missing/incomplete data: BMI (*n* = 46), lipid variables (*n* = 2519), and implausible values of BMI (< 15 or > 50 kg/m^2^) = 876 were excluded. Again, respondents with no data on lipid-lowering drugs (41,156) were excluded. Hence, 135,403 weighted participants were finally used for the analysis.

Continuous variables were presented as mean ± standard deviation (SD) for normal distribution, and median and interquartile range (IQR) for skewed distribution. Categorical variables were reported as numbers (percentages), and by proportions and 95% confidence intervals (CIs). The awareness, treatment, and control rates were standardized to the age- and sex-specific structure of the 2010 Chinese national population census. We explored the associations between determinants (categorical) and the outcome variables of interest (awareness, treatment, and control), using univariate and multivariable logistic models. Data were presented as crude odds ratios (COR) and adjusted odds ratios (AOR) with 95% CI. All statistical tests were two-tailed and p-values ≤ 0.05 were considered statistically significant. Processing of graphs and tables was done using Microsoft (MS) excel 2013.

### Ethical statement

This study was approved by the Ethics Committee of the Xuanwu Hospital Institutional Review Board, Capital Medical University (Beijing, China), and performed according to the declaration of Helsinki. Informed consent was obtained from all survey participants.

## Results

### Participants

Table 1 summarizes the description of participants’ characteristics according to their dyslipidemia status. Overall, 51.1% of subjects were women. The overall mean age ± SD was 56.6 ± 9.9 years. More than one-third of respondents were aged 50–59 years (42.3%). Approximately three-fifths (62.1%) of the subjects had primary or no education, and over 50% lived in rural areas (53.6%). Majority of the participants (94.3%) had a family history of dyslipidemia. Slightly over 40% of them were overweight, about 33% were current smokers, 19% drank alcohol, 62% were physically active, and about 58% had central obesity.

**Table 1:** Participants' characteristics and dyslipidemia status.

Variables	Total *N* (%)	Dyslipidemia *N* (%)	Normal *N* (%)	*p-value
Total	135,403	57,760 (42.7)	77,642 (57.3)	
Age (years)	56.6 ± 9.9	56.5 ± 9.7	56.6 ± 10.2	0.120
**Median (IQR)**				
40–49	35,617 (26.3)	14,821 (25.7)	20,796 (26.8)	< 0.001
50–59	57,300 (42.3)	25,037 (43.3)	32,263 (41.6)	
60–69	26,154 (19.3)	11,437 (19.8)	14,717 (19.0)	
70 and above	16,331 (12.1)	6465 (11.2)	9866 (12.7)	
**Sex**				
Men	66,259 (48.9)	27,816 (48.2)	38,443 (49.5)	< 0.001
Women	69,144 (51.1)	29,945 (51.8)	39,199 (50.5)	
**Residence**				
Rural	72,556 (53.6)	31,292 (54.2)	41,264 (53.1)	< 0.001
Urban	62,847 (46.4)	26,469 (45.8)	36,378 (46.9)	
**Nationality**				
Han	130,934 (96.7)	55,557 (96.2)	75,377 (97.1)	< 0.001
Others	4465 (3.3)	22.3 (3.8)	2262 (2.9)	
**Marital status**				
Married	80,154 (59.2)	32,362 (56.0)	47,792 (61.6)	< 0.001
Unmarried	55,248 (40.8)	25,398 (44.0)	29,850 (38.4)	
**Level of education**				
Primary and below	84,118 (62.1)	37,459 (64.9)	46,659 (60.1)	< 0.001
Junior/Middle School	32,233 (23.8)	12,264 (21.2)	19,969 (25.7)	
Senior High School	13,104 (9.7)	5598 (9.7)	7506 (9.7)	
College and above	5948 (4.4)	2440 (4.2)	3508 (4.5)	
**Geographical regions**				
North	75,174 (55.5)	29,172 (50.5)	46,002 (59.2)	< 0.001
South	60,229 (44.5)	28,589 (49.5)	31,640 (40.8)	
**Stroke belt zone**				
Yes	23,262 (17.2)	9449 (16.4)	13,813 (17.8)	< 0.001
No	112,142 (82.8)	48,312 (83.6)	63,830 (82.2)	
Weight	65.0 (58.0–71.5)	65.0 (58.0–72.0)	55.0 (49.0–63.0)	< 0.001
Height	162.1 ± 8.4	161.9 ± 8.6	162.2 ± 8.3	< 0.001
**BMI**				
Median (IQR)	25.2 (23.0–27.5)	25.7 (23.5–27.9)	25.1 (22.7–27.1)	< 0.001
Underweight (< 18.5 kg/m^2^)	46,598 (34.4)	16,968 (29.4)	29,630 (38.2)	< 0.001
Normal (≥ 18.5 to < 24.0 kg/m^2^)	1835 (1.4)	568 (1.0)	1267 (1.6)	
Overweight (≥ 24.0 to < 28 kg/m^2^)	59,314 (43.8)	26,232 (45.4)	33,082 (42.6)	
Obesity (≥ 28.0 kg/m^2^)	27,655 (20.4	13,992 (24.2)	13,663 (17.6)	
**Waist circumference**^**†**^				
Median (IQR)	86.0 (80.0–93.0)	88.0 (80.0–95.0)	85.0 (79.0–92.0)	< 0.001
Normal ≤ 90/80 cm (M/W)	56,214 (42.0)	21,034 (36.7)	35,180 (45.9)	< 0.001
Central obese ˃ 90/80 cm (M/W)	77,700 (58.0)	36,230 (63.3)	41,470 (54.1)	< 0.001
Family history of dyslipidemia	127,624 (94.3)	53,695 (93.0)	3714 (4.8)	< 0.001
Drinking (current)	25,747 (19.0)	11,249 (19.5)	14,498 (18.7)	< 0.001
Smoking (current)	43,992 (32.5)	19,078 (33.0)	24,914 (32.1)	< 0.001
Physical activity	83,913 (62.0)	35,349 (61.2)	48,564 (62.5)	< 0.001
**Blood lipids (mmol/l), median (IQR)**				
TC	4.9 (4.2–5.6)	5.3(4.4–6.3)	4.7 (4.2–5.3)	< 0.001
HDL-C	1.3 (1.1–1.6)	1.1 (0.9–1.4)	1.4 (1.2–1.7)	< 0.001
LDL-C	2.9(2.2–3.4)	3.0 (2.2–3.9)	2.8 (2.2–3.2)	< 0.001
TG	1.5 (1.1–2.2)	2.3 (1.5–3.1)	5.5 (5.0–6.2)	< 0.001
Non-HDL-C	3.5 (2.8–4.2)	4.1 (3.3–4.9)	3.2 (2.6–3.8)	< 0.001

Dyslipidemia was defined as total cholesterol (TC) > 6.22 mmol/L or low-density lipoprotein (LDL-C) > 4.14 mmol/L or high-density lipoprotein (HDL-C) < 1.04 mmol/L or triglycerides (TG) > 2.26 mmol/L or non-high density lipoprotein > 4.9 mmol/L or self-reported treatment. Data shown as mean ± SD, or median (IQR) or n (%). *p-value for comparison of dyslipidemia vs. normal subjects. Men (M), Women (W). Unmarried (widowed/divorced/others).

### Awareness, treatment, and control of dyslipidemia

In this study, we found that, 42.7% of Chinese adults had dyslipidemia. Among them, 64.0% were aware of their condition, 18.9% received treatment, and 7.2% had adequately controlled dyslipidemia.

Participants aged ≥ 70 years, and 60–69 years recorded the highest proportion for awareness (64.3%), and for treatment (19.2%), respectively. Adequately controlled dyslipidemia was highest among the 40–49 year age group with (9.8%). Women compared with men had higher proportion of awareness, treatment, and control (69.2% vs. 58.3%, 23.6% vs. 12.9%, and 8.3% vs. 4.7%, respectively; p < 0.001). See Table 2 for further details**.**

**Table 2:** Awareness, treatment, and control of dyslipidemia among subjects.

Variable		Awareness	Treatment	Control
Total		36,958 (64%)	6993 (18.9%)	504 (7.2%)
Age group (years)	40–49	64.2 (63.4–64.9)	19.1 (12.3–27.6)	9.8 (5.1–16.8)
	50–59	63.7 (63.1–64.3)	18.8 (12.1–27.3)	6.8 (3.1–13. 0)
	60–69	64.2 (63.3–65.1)	19.2 (12.4–27.7)	5.2 (2.1–10.9)
	70 and above	64.3 (63.1–65.4)	18.3 (11.7–26.7)	6.6 (2.9–12.7)
		p = 0.718	p = 0.632	p < 0.001
Sex	Men	58.3 (57.8–58.9)	12.9 (7.4–20.5)	4.7 (1.8–10.2)
	Women	69.2 (68.7–69.7)	23.6 (16.1–32.6)	8.3 (4.1––14.9)
		p < 0.001	p < 0.001	p < 0.001
Residence	Rural	61.0 (60.5–61.6)	18.6 (11.9–27.1)	8.2 (4.0–14.8)
	Urban	67.5 (66.9–68.1)	19.3 (12.5–27.8)	6.2 (2.7–12.2)
		p < 0.001	p = 0.081	p < 0.001
Ethnicity	Han	67.5 (66.9–68.1 )	19.0 (12.3–27.5)	7.3 (3.4–13.6)
	Others	61. 0 (60.5–61.6)	16.8 (10.5–25.0)	4.3 (1.5–9.6)
		p < 0.001	p = 0.040	p = 0.077
Marital Status	Married	63.8 (63.3–64.3)	19.5 (12.7–28.1)	7.0 (3.2–13.3)
	Unmarried	64.2 (63.6–68.2)	18.2 (11.6–26.6)	7.5 (3.5–13.9)
		p = 0.367	p < 0.001	p = 0.0.371
Level of education	Primary and below	62.6 (62.1–63.1)	18.4 (11.8–26.8)	7.3 (3.4–13.6)
	Junior/Middle School	65.6 (64.8–66.4)	20.1 (13.2–28.7)	7.2 (3.3–13.5)
	Senior High School	67.0 (65.8–69.8)	19.3 (12.5–27.8)	6.7 (3.0–12.9)
	College and above	69.5 (67.7–71.3)	19.6 (12.7–28.2)	7.2 (3.3–13.5)
		p < 0.001	p = 0.001	p = 0.961
Geographical regions	North	63.1 (53.4–72.1)	19.5 (12.7–28.1)	8.7 (4.3–15.4)
	South	64.8 (55.1–73.6)	18.4 (11.8–26.8)	5.6 (2.3–11.4)
		p < 0.001	p = 0.009	p < 0.001
Stroke belt	Yes	64.0 (54.3–72.9)	18.0 (11.4–26.4)	5.0 (1.9–10.6)
	No	64.8 (55.1–73.6)	19.1 (12.3–27.6)	7.6 (3.6–14.0)
		p < 0.001	p = 0.069	p = 0.003
BMI	Underweight (< 18.5 kg/m^2^)	64.6 (63.9–65.3)	18.3 (11.7–26.7)	8.5 (4.2–15.2)
	Normal (≥ 18.5 to < 24.0 kg/m^2^)	62.1 (58.1–66.1)	14.4 (8.6–22.3)	7.8 (3.7–14.3)
	Overweight (≥ 24.0 to < 28 kg/m^2^)	63.6 (63.0–64.2)	19.2 (12.4–27.7)	6.9 (3.1–13.1)
	Obesity (≥ 28.0 kg/m^2^)	64.0 (63.2–64.8)	19.4 (12.6–28.0)	6.4 (2.8–12.5)
		p = 0.179	p = 0.032	p = 0.075
Waist circumference	Normal ≤ 90/80 cm	62.3 (52.6–71.3)	17.7 (11.2–26.0)	7.3 (3.4–13.6)
	Central obese ˃ 90/80 cm	64.9 (55.6–73.7)	19.7 (12.8–28.3)	7.1 (3.3–13.4)
		p < 0.001	p < 0.001	p < 0.001
Family history of dyslipidemia	Yes	66.2 (56.6–74.9)	18.9 (12.2–27.4)	7.3 (3.4–13.6)
	No	34.6 (25.8–44.3)	19. 7 (12.8–28.3)	4.3 (1.5–9.6)
		p < 0.001	p = 0.438	p = 0.061
Drinking (current)	Yes	68.6 (59.1–77.1)	68.6 (59.1–77.1)	6.3 (2.7–12.3)
	No	62.9 (53.2–71.9)	62.9 (53.2–71.9)	7.6 (3.6–14.0)
		p < 0.001	p < 0.001	p = 0.058
Smoking (current)	Yes	68.6 (59.1–77.1)	68.6 (59.1–77.1)	7.6 (3.6–14.0)
	No	61.7 (51.9–70.8)	61.7 (51.9–70.8)	6.9 (3.1–13.1)
		p < 0.001	p < 0.001	p = 0.279
Physical activity	Yes	64.1 (54.4–73.0)	64.1 (54.4–73.0)	7.6 (3.6–14.0)
	No	63.8 (54.1–72.7)	63.8 (54.1–72.7)	6.6 (2.9–12.7)
		p = 0.370	p = 0.370	p = 0.100

Proportion in each column has been calculated using the values in previous column as a denominator, unmarried (widowed/divorced/others).

**Figure 1:**
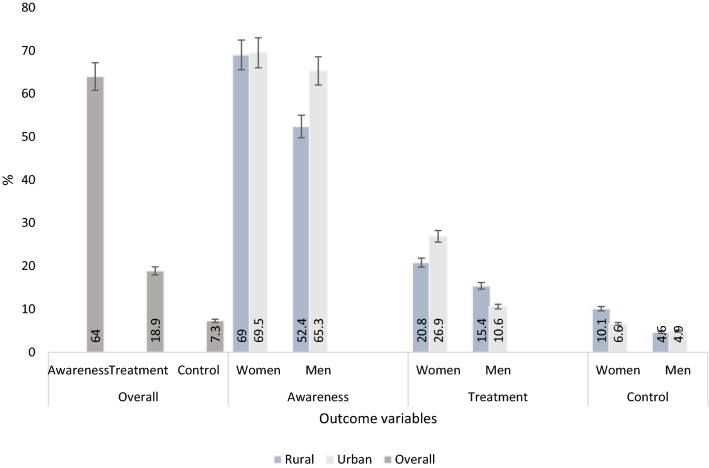
The overall, sex and rural/urban proportions of dyslipidemia awareness, treatment and control among the study population.

The awareness, treatment, and control of dyslipidemia according to sex and place of residence are shown in Fig. 1. The percentage of dyslipidemia awareness among women in both areas of residence- rural in rural and urban settings was similar (p > 0.05). Whereas, urban men recorded higher awareness rate compared to their rural counterparts. Treatment was higher in men living in rural compared to urban areas. On the other hand, urban women compared with their rural counterparts had greater treatment rate. Dyslipidemia control was higher in women than in men of both places of residence (all p < 0.05).

### Determinants of awareness, treatment, and control of dyslipidemia

Table 3 shows the CORs and AORs of dyslipidemia determinants. In the multivariable logistic regression analysis, the likelihood of dyslipidemia awareness was higher in women compared with men (AOR 1.14, 95% CI 1.11–1.18). The results further indicated that urban residents, current drinkers, obese persons, and living in northern China were positively associated with dyslipidemia awareness, and current smokers and living in the stroke belt zone were negatively associated with dyslipidemia awareness (all p < 0.05). The awareness of dyslipidemia was more than three times greater in adults with a family history of dyslipidemia in their immediate family compared to those without (AOR 3.91, 95% CI 3.72–4.01).

**Table 3:** Univariate and multivariable analyses of factors affecting awareness, treatment, and control of dyslipidemia among adults.

Variable			Awareness		Treatment		Control	
			COR (95% CI)	AOR (95% CI)^a^	COR (95% CI)	AOR (95% CI)^a^	COR (95% CI)	AOR (95% CI)^a^
Age		50–59	0.98 (0.94–1.02)		0.99 (0.94–1.05)		0.67 (0.54–0.82)	0.69 (0.55–0.85)
	(Ref: 40–49)	60–69	1.00 (0.95–1.05)		1.02 (0.96–1.09)		0.51 (0.38–0.67)	0.54 (0.40–0.71)
		70 and above	1.00 (0.94–1.07)		0.98 (0.91–1.06)		0.64 (0.47–0.89)	0.68 (0.49–0.95)
Sex	(Ref: men)		1.61 (1.55–1.66)	1.14 (1.11–1.18)	1.89 (1.81–1.97)	2.01 (1.88–2.15)	1.83 (1.46–2.29)	1.73 (1.37–2.18)
Residence	(Ref: rural)		1.33 (1.28–1.37)	1.15(1.12–1.18)	1.00 (0.96–1.05)		0.74 (0.62–0.89)	0.69 (0.57–0.83)
Nationality	(Ref: others)		1.07 (0.95–1.13)		1.07 (0.96–1.20)		1.74 (0.92–3.28)	
Marital Status	(Ref: unmarried)		0.98 (0.95–1.02)		1.06 (1.02–1.11)	1.07(1.01–1.13)	0.92 (0.77–1.10)	
Level of Education		Junior/Middle School	1.14 (1.09–1.19)	0.99 (0.96–1.01)	1.10 (1.04–1.16)	1.03 (0.96–1.10)	0.98 (0.79–1.22)	
	(Ref: primary and below)	Senior High School	1.21 (1.14–1.29)	0.97 (0.93–1.01)	1.07 (1.00–1.15)	0.98 (0.89–1.07)	0.92 (0.67–1.25)	
		College and above	1.36 (1.25–1.49)	1.02 (0.96–1.09)	1.15 (1.03–1.27)	0.92 (0.81–1.06)	0.99 (0.64–1.52)	
Geographical regions	(Ref: South)		0.93 (0.90–0.96)	1.05 (1.02–1.08)	1.02 (0.98–1.06)		1.59 (1.32–1.92)	1.91 (1.57–2.32)
Stroke belt	(Ref: Non-stroke belt)		0.80 (0.77–0.84)	0.85 (0.83–0.88)	0.91 (0.86–0.97)	0.93 (0.87–1.01)	0.64 (0.47–0.86)	0.52 (0.38–0.71)
BMI		Underweight (< 18.5 kg/m^2^_)_	0.90 (0.76–1.07)	0.94 (0.85–1.04)	0.84 (0.67–1.06)	0.78 (0.58–1.01)	0.94 (0.34–2.62)	1.21 (0.43–3.43)
	(Ref: Normal (≥ 18.5–< 24.0 kg/m^2^)	Overweight (≥ 24.0–< 28 kg/m^2^)	0.96 (0.92–0.99)	1.00 (0.97–1.03)	1.07 (1.02–1.13)	1.03 (0.96–1.10)	0.80 (0.65–0.99)	0.79 (0.64–0.96)
		Obesity (≥ 28.0 kg/m^2^)	0.98 (0.93–1.02)	1.04(1.00–1.07)	1.09 (1.03–1.15)	1.06 (0.98–1.14)	0.74 (0.58–0.95)	0.74 (0.58–0.95)
Waist circumference	(Ref: Normal ≤ 90/80 cm)		1.12 (1.08–1.16)	1.01 (0.98–1.03)	1.15 (1.10–1.20)	0.96 (0.90–1.02)	0.96 (0.80–1. 17)	
Family history of dyslipidemia	(Ref: no)		3.71 (3.47–3.97)	3.91 (3.72–4.01)	0.94 (0.87–1.02)		1.67 (0.94–2.98)	
Drinking (current)	(Ref: no)		1.29 (1.24–1.35)	1.06 (1.03–1.10)	1.53 (1.46–1.61)	1.18 (1.10–1.26)	0.81 (0.66–1.01)	
Smoking (current)	(Ref: no)		1.36 (1.31–1.41)	0.95 (0.92–0.98)	1.42 (1.36–1.48)	0.96 (0.90–1.02)	1.11 (0.92–1.33)	
Physical activity	(Ref: no)		1.02 (0.98–1.05)		1.02 (0.98–1.07)		1.17 (0.97–1.42)	

*COR* Crude odds ratio, *AOR* adjusted odds ratio, *CI* confidence interval. Unmarried (widowed/divorced/others).

^a^Adjusted for all variables cited in the table.

Among those who were aware they had dyslipidemia, positive association with treatment was seen among women compared to men, married compared to unmarried respondents, and alcohol drinkers compared to non-drinkers (all p < 0.05).

For subjects who had been treated for dyslipidemia previously, the results showed that women and persons living in northern China were positively associated with dyslipidemia control. Whereas, the probability of control decreased among individuals living in the stroke belt zone and those with more than normal body weight (overweight and obesity). The likelihood of control was 31% lower in urban compared with rural participants (AOR 0.69, 95% CI 0.57–0.83). See Table 3 for further details**.**

## Discussion

This study estimated the awareness, treatment, and control of dyslipidemia and their determinants among middle-aged and older Chinese adults using a nationwide survey data. Our findings revealed that 64% of the subjects had dyslipidemia, of whom 18.9% received treatment, and of whom 7.2% had adequately controlled lipids. Treatment and control rates in both places of residence were higher in women than in men. The multivariable analysis revealed that women, urban residents, positive family history of dyslipidemia, current drinking, and general obesity had increased odds of awareness. In addition, women, married respondents, and current drinkers had higher odds of treatment. Age group, overweight, general obesity, stroke belt zone, urban residence, and women were independent determinants of control.

“The rule of halves” framework^10^ suggests that almost 50% of patients should be aware of their disease, of whom about 50% should receive treatment, and of whom about 50% achieve should treatment targets. In the case of our respondents, 64% were aware of their disease status, which suggests desirable level of awareness. Yet, the treatment (18.9%) and control (7.2%) rates were far below expected levels. However, compared with some previous reports, the current findings demonstrated improvements in dyslipidemia awareness, treatment, and control rates in China^9,26,27^. The significance of the present study has been emphasized by the low treatment and control rates found. These low rates may signify some gaps and shortcomings such as poor adherence to medications^28^, and drug prescription challenges^29,30^ associated with the current dyslipidemia management strategy. These findings may also support the speculation that providers were not very effective at targeting treatment to goal. Thus, a comprehensive dyslipidemia management approach needs to be emphasized. In addition to the use of pharmacological approach, it is essential to focus on all strategies embedded in the levels of prevention, including targeted and opportunistic screenings, health promotion programs on lifestyle modifications and good nutrition for effective and efficient management as drug therapy alone does not control dyslipidemia completely.

In the present study, subjects aged 40–49 years had higher odds of dyslipidemia control compared with their older counterparts. This result is partly similar to a Korean study where younger adults were more likely to have controlled LDL-C^31^. However, many contradictory results have been reported^8,26,29,32^. Our results may support the finding that control rates of dyslipidemias such as raised TC may differ depending on certain participants’ characteristics and country of origin, and rates may range from 18 to 100%^33^.

Consistent with prior reports^26,29^, our study found that women were more likely to be aware of and treated for dyslipidemia, and they were more likely to have their condition adequately controlled compared to men. Similarly, two studies on the management of raised LDL-C recorded higher likelihood of awareness, treatment, and control among women^4,34^. Behavioral differences between the sexes could partly explain this result, as women are known to seek healthcare services more often than men^4^. Hence, we suggest that men should be considered as a special group of interest in dyslipidemia management in this country.

In line with our results, an earlier study^19^ demonstrated a positive relationship between dyslipidemia awareness and urban residence. Similarly, a study in Thailand found high awareness rate for raised LDL-C among urban residents^35^. Further, a couple of findings from low- and middle-income countries reported lower dyslipidemia awareness and treatment rates in rural settings^22,36^. The high level of awareness found in urban areas in the present study may be attributed to the wealthier and better educated populations usually found in cities and municipalities^37^. Again, it may buttress the evidence of difficult access to health care common in rural areas^13^. So, health promotion programs should target deprived areas with limited resources. Surprisingly, lower level of dyslipidemia control was seen among urban subjects.

The result of our study shows that married persons were more likely to receive treatment for their condition. This finding is in line with another study on the treatment of hypertension^38^. According to the social causation theory, individuals could benefit from spousal support^39^. For example, living together allows quicker appreciation and response to threatening signals. Again, spouses, mostly wives, usually encourage the adoption of healthy behavior such as a healthy lifestyle and adherence to treatment that may promote cardiovascular health^40^.

From this study, staying in northern China was an independent determinant of dyslipidemia awareness and control. A previous study indicated that stroke prevalence was significantly higher in the northern parts of China^20^, and living in the north was associated with high prevalence of dyslipidemia^7,41^. We speculate that the high prevalence of stroke and dyslipidemia found in the north could positively affect inhabitants’ behaviors towards treatment and result in favorable outcomes.

The role of geographic determinants in CVD risk factors’ control is essential for developing effective population-wide clinical and preventive strategies^42^. In the current study, individuals living in the stroke belt zone had lower likelihood of dyslipidemia control. This is inconsistent with the results of the Geographic and Racial Differences in Stroke (REGARDS) study, which reported lower likelihood of dyslipidemia control outside the stroke belt zone^43^. The reason for the current finding could be that effective and efficient management of lipid disorders may not be related to the stroke belt zone. Further studies are required to investigate why control of dyslipidemia were less likely in the stroke belt zone.

We observed a positive association between dyslipidemia awareness and general obesity. This is consistent with an earlier study^19^, but contrary to another report^44^. In addition, dyslipidemia awareness relates to weight management. It has been noted that awareness can improve weight perception and management since obesity is a risk factor of dyslipidemia and weight loss has beneficial effects on dyslipidemia prevention and control^45^. Also, we found general obesity as a significant negative predictor of dyslipidemia control, which is similar to earlier findings^8,19^. A probable explanation could be that dyslipidemia control is difficult in individuals with more than normal body weight.

Persons with a family history of dyslipidemia in their immediate family had the strongest independent association of awareness in this study. This is in line with the result of He et al.^8^, where persons with a family history of dyslipidemia had higher likelihood of awareness. Predictably, family members would be more mindful and watchful of the condition and doctors would pay more attention to these patients. This study found that current drinking was positively associated with dyslipidemia awareness and treatment. This was dissimilar to a study in the Jilin Province^8^. The reason for the current finding could be that individuals with dyslipidemia who are alcohol drinkers might usually receive counselling on drinking cessation and treatment from physicians or other health workers, so they are more likely to be aware and treated for lipid disorders. Current smokers were less likely to get their condition treated in the current study. This is in accordance with an earlier report in China^8^. This result could mean that smokers don’t pay enough attention to their healthcare^46^.

### Strengths and limitations

Key strengths of the study include its population-based design, representative of various urban and rural communities across China. Thus, the nationwide coverage allows for calculation of nationally representative estimates. Further, the analysis consisted of many determinants of dyslipidemia awareness, treatment, and control, which may provide a broader view of the relative role of different determinants in prevention efforts.

This survey was cross-sectional; thus, the opportunity to explore casualty was limited. Another limitation observed was that participants’ previous experience with medication use for other conditions could affect treatment adherence. Also, we did not investigate the effect of cognitive factors such as self-efficacy and illness perception of people who received treatment and had controlled dyslipidemia. Information bias might occur from self-reported characteristics.

## Conclusion

In summary, we found high dyslipidemia awareness rate, with low treatment and control rates. Higher proportions of awareness, treatment, and control were noted in women than in men. Treatment rate was higher in rural than urban men, but women in urban areas showed higher treatment rate compared with their rural counterparts. Dyslipidemia control rate was greater in women than men in both rural and urban areas.

Women, urban residence, positive family history of dyslipidemia, and general obesity were positively associated with awareness. Married respondents, women, and current drinkers were more likely to be treated for dyslipidemia. Age groups, general obesity, women, urban residence, and overweight had significant relationship with control. Therefore, improved health education and treatment are needed for better dyslipidemia management.

## Abbreviations

CNSSPP: China National Stroke Screening and Prevention Project
CVD: Cardiovascular disease
TC: Raised total cholesterol
TG: Raised triglycerides
LDL-C: Raised low-density lipoprotein
HDL-C: Low high-density lipoprotein
